# Nociceptor α7nAChR activation blunts neuronal HMGB1 release and attenuates inflammation and nociceptive behavior

**DOI:** 10.1186/s10020-025-01387-z

**Published:** 2025-11-03

**Authors:** Huan Yang, Timothy S. Morgan, Serena Petruzzelli, Okito Hashimoto, Tyler D. Hepler, Aisling Tynan, Saher Chaudhry, Michael Brines, Ulf Andersson, Sangeeta S. Chavan, Kevin J. Tracey

**Affiliations:** 1 Institute for Bioelectronic Medicine, Feinstein Institutes for Medical Research, 350 Community Drive, Manhasset, NY 11030 United States; 2 Department of Women’s and Children’s Health, Karolinska Institute, Karolinska University Hospital, Stockholm, 17176 Sweden; 3 Elmezzi Graduate School of Molecular Medicine, Feinstein Institute for Medical Research, Northwell Health, Manhasset, NY 11030 United States; 4 Donald and Barbara Zucker School of Medicine at Hofstra/Northwell, Hempstead, NY 11549 United States

**Keywords:** HMGB1, α7 nicotinic acetylcholine receptor, Cholinergic anti-inflammatory pathway, Inflammation, Pain-like behavior, Dorsal root ganglia, Cholinergic agonists

## Abstract

**Background:**

High Mobility Group Box 1 (HMGB1) is a nuclear protein that upon extracellular release acts as an alarmin to initiate and amplify inflammation. HMGB1 release from nociceptors contributes to both inflammation and pain; however, the mechanisms for its regulation remain incompletely understood. The cholinergic anti-inflammatory pathway, mediated by α7 nicotinic acetylcholine receptor (α7nAChR) activation, inhibits HMGB1 release from immune cells and reduces inflammation. This study investigates whether α7nAChR signaling similarly inhibits HMGB1 release from nociceptors, thereby affecting pain and inflammation.

**Methods:**

Dorsal root ganglia (DRG) neurons were isolated from C57BL/6 or VGlut2-Cre/ChR2-YFP mice (expressing ChR2 in sensory neurons for optogenetic stimulation at 470 nm). HMGB1 release in vitro was triggered by optogenetic stimulation or exposure to capsaicin (5 µM), in the presence or absence of cholinergic agonists (acetylcholine, GTS-21, PNU-282987), and subsequently measured by ELISA. Immunohistochemistry was used to visualize cellular HMGB1 localization. In vivo models, including optogenetic stimulation and formalin-induced pain-like behavior, were used to evaluate the effects of cholinergic agonists on pain-like behavior, mechanical allodynia and inflammation. α7nAChR knockout (KO) mice served to determine receptor-specific effects. Levels of proinflammatory mediators calcitonin gene-related peptide (CGRP), substance P, HMGB1, and IL-6 were also measured.

**Results:**

Optogenetic stimulation of cultured DRG neurons significantly increased HMGB1 release, which was markedly inhibited by cholinergic agonists. Similarly, capsaicin-induced HMGB1 release was suppressed by acetylcholine, GTS-21, and PNU-282987, promoting HMGB1 retention within the nucleus; this effect was abolished in α7nAChR KO neurons. In contrast, the release of CGRP and substance P following optogenetic or capsaicin stimulation of DRG neurons from wild-type mice was not influenced by cholinergic agonists. In vivo, GTS-21 reduced pain-like behaviors and mechanical allodynia in both the formalin-induced and optogenetically-stimulated nociceptive behavior models, as demonstrated by reduced mechanical allodynia and extracellular HMGB1 levels. These effects were absent in α7nAChR KO mice, confirming the critical role of α7nAChR in mediating these responses.

**Conclusion:**

This study reveals a novel α7nAChR-dependent cholinergic mechanism that reduces nociceptive behavior and inflammation by retaining nuclear HMGB1 in nociceptors. Cholinergic agonists may serve as promising therapeutic agents to mitigate nociceptive behavior and inflammation by targeting α7nAChR in sensory neurons.

**Supplementary Information:**

The online version contains supplementary material available at 10.1186/s10020-025-01387-z.

## Introduction

High Mobility Group Box 1 (HMGB1) is a nuclear chromatin-binding protein that, upon translocation to the extracellular environment, functions as a damage-associated molecular pattern (DAMP), playing a critical role in inflammation (Andersson et al. 2018a; Chen et al. 2020; Deng 2018; Kang et al. 2014; Yang et al. 2020; Yang 2015). Extracellular HMGB1 acts as a chemoattractant or pro-inflammatory mediator, stimulating immune cells to release cytokines such as tumor necrosis factor (TNF), interleukin (IL)−1, and IL-6, thereby initiating an inflammatory response (Andersson et al. 2000, 2018b).

The cholinergic anti-inflammatory pathway is a neural mechanism mediated by the vagus nerve that protects against dysregulated systemic inflammation. Vagus nerve activation releases acetylcholine, which signals via the α7nAChR, expressed by macrophages, lymphocytes, neurons and other cells, to inhibit pro-inflammatory cytokine release which protects against systemic inflammation (Andersson 2020; Gauthier et al. 2021; Tracey 2002; Wang et al. 2004; Zaghloul et al. 2017). Over the years, we and others have demonstrated that therapies using cholinergic agonists are effective in reducing systemic HMGB1 levels and inhibiting inflammation in many preclinical and clinical disease models (Chen et al. 2022; Huston et al. 2007a; Li et al. 2013, 2016; Lu et al. 2014a; Naser and Kuner 2018; Saeed et al. 2005; Sitapara et al. 2020a; Tsoyi et al. 2011; Wang et al. 2004; Yang et al. 2019; Yu et al. 2013; Zhang et al. 2019, 2023; Zhou et al. 2021; Zi et al. 2019). Additionally, we have observed similar beneficial effects from cholinergic agonists and anti-HMGB1 antibodies in inflammatory disease models (Andersson 2020; Deng et al. 2022; Mao et al. 2022; Wang et al. 2004).

Despite substantial evidence indicating the critical role of cholinergic regulation in inflammation and reducing HMGB1 levels, some of the underlying mechanisms in inflammation have remained unclear. Most prior studies focused on cholinergic effects mediated directly on immune cells. However, recent findings revealed that HMGB1 can also be actively released from neurons (Yang 2021). In nerve injury models such as chronic constriction injury (CCI) or collagen antibody-induced arthritis (CAIA), neurons are the source of HMGB1, leading to inflammation and nociceptive behavior. Thus, neuronal HMGB1 knockout (KO) mice are protected from collagen antibody-induced arthritis and sciatic nerve injury-induced allodynia (Yang 2021). These findings strongly indicate that neuron-derived HMGB1 plays a crucial role in inflammation and nociceptive behavior; however, the mechanisms for its regulation remain unclear. Since α7nAChR is expressed by DRG neurons (Shelukhina et al. 2009), we reasoned that cholinergic signaling via α7nAChR may regulate HMGB1 release also from sensory neurons.

## Materials and methods

### Materials

Poly-L lysine and laminin, capsaicin, formalin, acetylcholine chloride, pyridostigmine bromide, α7nAChR selective agonist GTS-21, EGA (Cat# SML1006), 3-isobutyl-1-methylxanthine (IBMX), anti-β actin monoclonal antibody (Cat # SAB5500001), nerve growth factor (NGF, Cat # N6009), laminin (Cat # L-2020) and cytotoxicity detection kit (LDH) were purchased from Sigma-Aldrich (St. Louis, MO). The α7nAChR selective agonist PNU-282987 was obtained from Tocris Bioscience (Minneapolis, MN). Fetal bovine serum was sourced from Gibco BRL (Carlsbad, CA) and glutamine from Biowhittaker Inc., (Walkersville, MD). Protease inhibitor minitablets (Cat # A32953), neurobasal-A medium, neural growth factor, 1X B27 supplement, sterile nylon cell strainer (40 µm), penicillin and streptomycin, coverslip sealant (Cat # NC0154994) were obtained from Thermo Fisher Scientific Inc., (Waltham, MA). Collagenase/dispase II was purchased from Roche Applied Science (Indianapolis, IN), and 4% paraformaldehyde was from Santa Cruz Biotechnology (Cat # sc-281692, Santa Cruz, CA). Poly-D-lysine/laminin coated coverslips (12 mm) were from Corning (Bedford, MA). Normal rabbit, normal donkey serum (Cat# 0030–01) and Fluoromount mounting medium containing DAPI (4′,6- diamidino-2-phenylinodole) (Cat # 0100–20) were acquired from Southern Biotech (Birmingham, AL). Anti-NeuN antibody Alexa fluor-647 (Cat # ab190565, 1:200), monoclonal anti-HMGB1 antibody Alexa fluor 555 (Cat # ab206896, 1:500) were purchased from Abcam (Cambridge, MA). Donkey anti-chicken Alexa 488 was obtained from Invitrogen (Cat #A78948, 1:500, Carlsbad, CA).

### Animals

All procedures involving experimental animals were approved (IACUC protocol #2016–028) by the Institutional Animal Care and Use Committee and the Institutional Biosafety Committee of the Feinstein Institutes for Medical Research, Northwell Health, Manhasset, NY in accordance with NIH guidelines and the ethical guidelines of the international association for the study of pain. Animals were maintained at 25 °C on a 12-h light–dark cycle with free access to food and water. Sprague–Dawley rats (male, 2–3 months old), C57BL/6 mice (male, 2–3 months old), VGlut2-ires-Cre mice (Slc17a6tm2(cre)Lowl/J, male and female), ChR2-YFP-flox mice (B6.Cg-Gt(ROSA)26Sortm32(CAG-COP4*H134R/EYFP) Hze/J, male and female), and Acrα7 mice (B6.129S7-Chrna7tm1Bay/J, male and female) were obtained from Jackson Laboratories (Bar Harbor, ME). Animals were acclimated for 7 days prior to experimental use and housed under standard temperature and light–dark cycles. VGlut2-ires-Cre mice were bred with ChR2-YFP-flox mice to generate Vglut2-Cre/ChR2-YFP mice, which express the ChR2 allele under the control of the Vglut2 locus, encoding vesicular glutamate transporter type 2 that is predominantly expressed in peripheral sensory neurons. α7nAChR KO mice (Acrα7 mice) (B6.129S7-Chrna7tm1Bay/J) were bred in-house using Acrα7 parents. The genotypes of the transgenic strains were confirmed using PCR (Transnetyx, Cordova, TN).

### Neuronal cultures

The L_1_ to L_6_ dorsal root ganglia (DRG) from adult C57BL/6 or Vglut2-Cre/ChR2-YFP or α7nAChR KO mice (8–12 weeks old) were dissected and dissociated in collagenase/dispase II (1 mg/ml) in Hanks’ balanced salt solution (HBSS) at 37⁰C for 90 min. The DRGs were triturated and the cells filtered using a 40 µm nylon cell strainer with centrifugation. After centrifugation, cell pellets were suspended in neurobasal-A medium, supplemented with neural growth factor (50 ng/ml), 1X B27 supplement, penicillin, and streptomycin. The cells were then plated (10,000–15,000 cells/well) on coverslips pre-coated with poly-L-lysine (100 µg/ml) and laminin (50 µg/ml) and allowed to adhere for 12 to 15 h at 37 °C with 5% CO_2_. The cells were used at 48 h post-plating.

### Stimulation of DRG neurons with optogenetics

Primary DRG neurons isolated from Vglut2-Cre/ChR2-YFP mice were cultured on poly-lysine and laminin coated chamber plates for 48 h. The cultured cells were then activated by light stimulation using 470 nm light delivered by a light emitting diode at 20 Hz, 10% duty cycle for 15 min (DCZ100, ThorLabs, Newton, New Jersey), either with or without acetylcholine, GTS-21 or PNU-282987 added. Our prior work using this model has shown that although the release of HMGB1 is delayed compared to that of the neuropeptides CGRP and Substance P, all three molecules plateau within 60 min (Yang et al. 2022; Yang 2021). Therefore, the cell supernatants were collected at the end of 120 min for analysis.

### Stimulation of DRG neurons with capsaicin with or without cholinergic agonists

Primary DRG neurons were isolated from C57BL/6 or α7nAChR KO mice and cultured in poly-lysine and laminin coated chamber plates for 48 h. The cells were stimulated with capsaicin (5 µM) either with or without acetylcholine (0.1–10 µM), GTS-21 (0.1–10 µM) or PNU-282987 (100 µM). This dose is chosen based on previous publications (doses-response and toxicity studies in vitro)(Iwamoto et al. 2014; Shen et al. 2024). Pyridostigmine bromide was added whenever acetylcholine was used to inhibit its degradation. Cell supernatants were collected 120 min post-stimulation for further analysis.

### Immunohistochemical staining of DRGs

DRGs (L1–L6) were isolated from C57Bl/6 or Vglut2-Cre/ChR2-YFP mice and cultured in plates with poly-L-lysine and laminin coated coverslips for 48 h before treatment with vehicle, capsaicin or capsaicin plus GTS-21 for 120 min. Post treatment, cultured DRGs were fixed with 4% paraformaldehyde and blocked with normal donkey serum for 1 h. Cells were incubated with rabbit anti-HMGB1 antibody Alexa Fluor-555, rabbit anti-NeuN Alexa Fluor-647 overnight at 4 °C, followed by donkey anti-chicken Alexa 488 overnight at 4 °C. After washing three times with PBS plus 0.1% Triton X-100, cells were cover-slipped with DAPI Fluoro-mount mounting medium. Images were acquired and analyzed using an LSM900 laser scanning confocal microscope (Carl Zeiss Microscopy, LLC., White Plains, NY). Data were analyzed and quantitated using Zen Blue software (Zeiss Microscopy).

### Quantification of nuclear HMGB1 translocation in immunofluorescence staining of DRGs

For quantification, 150 DRGs per group were randomly selected by a blinded operator from three representative 20 × tile scans (Hatayama et al. 2021). The HMGB1 positive (HMGB1 +) area within each DRG was traced using the spline tool in Zen Blue software. The traced areas were quantified and graphed for each group. Additionally, using the DAPI stain to identify DRG nuclei, DRGs exhibiting HMGB1 translocation were identified by the presence of HMGB1 + staining outside the nucleus, and the percentage of such DRGs was calculated for each group.

### Optogenetic stimulation of mice

Vglut2-Cre/ChR2-YFP mice were randomly assigned to each group and briefly anesthetized with isoflurane (1.5–2%) and stimulated on the plantar surface of the right hind paw at 470 nm LED light at (4.7 mW, 3 Hz, 20% duty cycle) for 15 min (Daou et al. 2013). Immediately following optogenetic stimulation, animals received an intraperitoneal (IP) injection of either GTS-21 (4 mg/kg) or PBS (control). Mechanical allodynia was measured 5 h post-stimulation with the investigator blinded to the condition. In some experiments, animals were euthanized, and the right hind paw was collected for biochemical analysis.

### EXPEL method

To extrude the interstitial fluid from paw tissue, an EXPEL methodology was adapted as previously described. (Costanza et al. 2018) Fresh paw tissue (about 0.5 g) was collected immediately after harvesting, cut into 3 mm pieces, and placed in a 10 ml syringe. 400 µl of hypertonic extraction buffer [500 ml PBS supplemented with 4.5 g NaCl and protease inhibitor (2X)] was added to the tissue. The plunger was then set to 5 ml line (allowing an intake of approximately 4 ml air-bubble), followed by alternating pressure for 1 min, by moving the plunger from 5 to 1 ml line, repeating this procedure for a total of 30 times for each sample. The EXPEL-extruded fluid was collected and stored at −20 °C for further analysis.

### Measurements of HMGB1, calcitonin gene-related peptide (CGRP), substance P and lactate dehydrogenase (LDH)

Levels of HMGB1 in the paw tissue or cell supernatants were quantitated using ELISA kits (IBL International, Hamburg, Germany). CGRP was measured using EIA kits (Cayman Chemical Company, Ann Arbor, MI), and substance P was quantified using EIA kits (R&D Systems, Minneapolis, MN). LDH content in the media was determined using a detection kit (Cayman Chemical Company, Ann Arbor, MI). Total protein content was measured using the Bradford assay (BioRad, Hercules, CA).

### Mechanical allodynia measurement

Mechanical allodynia was assessed using von Frey filaments and the Dixon up-down method (Chaplan 1994; Dixon 1980). Animals were allowed to acclimatize in the testing apparatus on a metal mesh floor for 30 min before testing. For assessment, the animal was placed on an elevated mesh platform, and filaments (exerting forces of 0.4–7.3 g, Ugo Basile, Varese, Italy) were inserted through the mesh to stimulate the plantar aspect of the hind paw in ascending order to define the threshold stimulus intensity required to elicit a paw withdrawal response. The filament was held in place for stimulation for approximately 5–7 s and was repeated for the injured paw after an interval of at least 5 min. The behavioral responses were used to calculate the absolute threshold (50% probability of response) as described previously (Milligan et al. 2000).

### Formalin-induced pain-like behavior model

C57BL/6 or α7nAChR KO mice (male, 8–12 weeks of age) were randomly assigned to each group and received an IP injection of GTS-21 (4 mg/kg) or vehicle 30 min prior to formalin injection. Formalin (5%, 10 µl/paw) was injected into the hind paw, and spontaneous pain-like behavior was assessed 5–45 min post-injection using the BASIC pain scoring system, with the person performing the test in a blinded fashion. The pain score program calculated, for each 5-min period, the time spent in four mutually exclusive categories of behavior: a pain score of 0 indicated normal weight bearing on the injected paw; a pain score of 1 indicated limping during locomotion or resting the paw lightly on the floor; a pain score of 2 indicated elevation of the injected paw so that at most the nails touched the floor; and a pain score of 3 indicated licking, biting, or grooming of the injected paw (Dubuisson and Dennis 1977). Additionally, Sprague–Dawley rats (male, 2–3 months old) randomly assigned to each group and received neutralizing anti-HMGB1 monoclonal antibody (2g7) (Yang et al. 2020) at 300 µg/rat IP prior to formalin (5%, 20 µl per hind paw), and pain-like behavior was assessed as above and again in a blinded manner.

### Statistical analysis

Data were analyzed using GraphPad Prism software. Normality was assessed using the Shapiro–Wilk test. Differences between treatment groups were determined by one-way ANOVA followed by Tukey’s multiple comparisons test. For comparison among multiple groups with non-normal (skewed) distribution, the Kruskal–Wallis ANOVA test followed by Dunn's post-hoc test was used to evaluate statistical differences. Student’s t test was used for comparison of two groups. P values less than 0.05 were considered statistically significant.

## Results

In light of previous findings demonstrating that cholinergic agonist-mediated signaling via α7nAChRs inhibits HMGB1 release from immune cells and ameliorates multiple inflammatory conditions (Chen et al. 2022; Huston et al. 2007b; Lu et al. 2014b; Naser and Kuner 2018; Pavlov et al. 2007; Sitapara et al. 2020b; Tsoyi et al. 2011; Wang et al. 2004; Zi et al. 2019), we used α7nAChR deficient mice to investigate whether the cholinergic agonists regulate HMGB1 release from nociceptors via an α7nAChR-dependent mechanism.

### GTS-21 ameliorated formalin-induced pain-like behavior in wildtype but not in α7nAChR KO mice

**Fig. 1:**
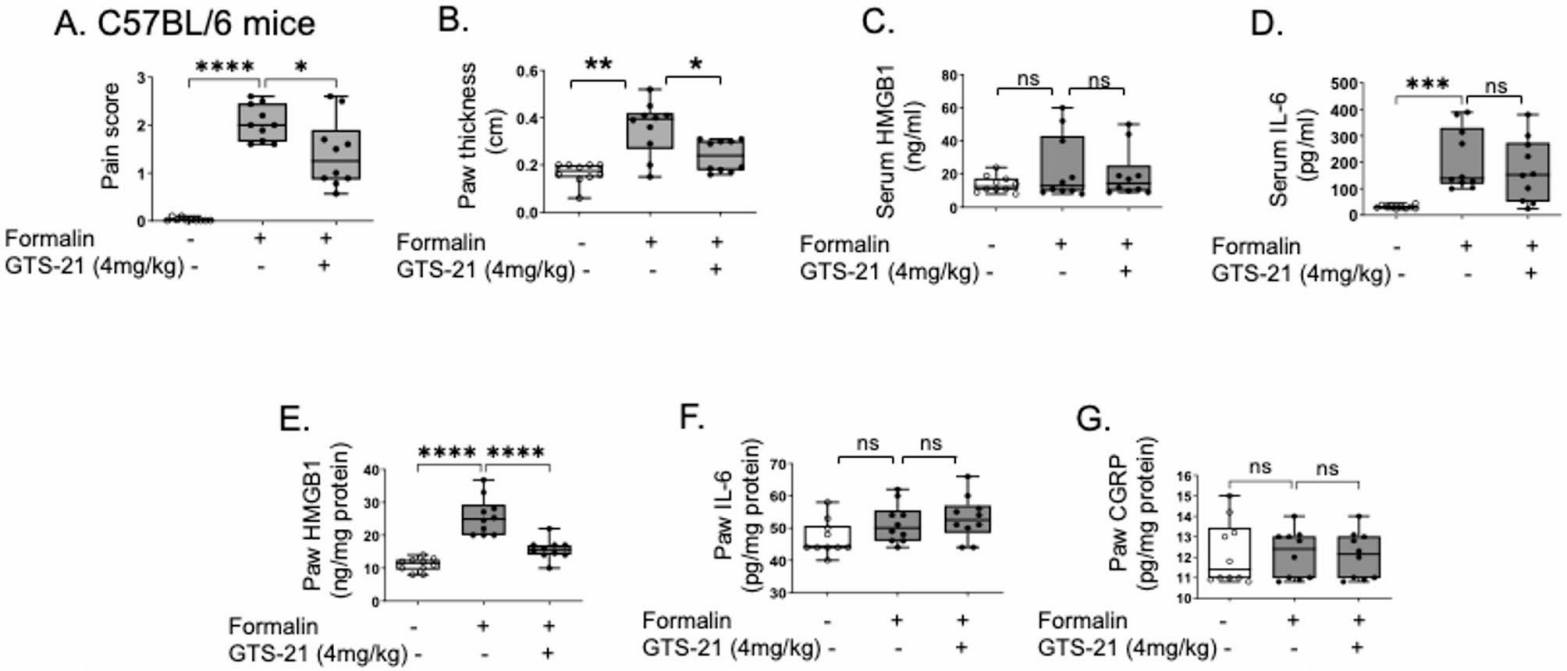
GTS-21 ameliorated formalin-induced pain-like behavior in C57BL/6 mice. C57BL/6 mice were administered GTS-21 (4 mg/kg) or vehicle (PBS) intraperitoneally (IP) 30 min before formalin injection (5%, 10 µl/paw) into the right hind paw. **A** Pain-like behavior was assessed 5 to 45 min post-injection. **B** Paw thickness was measured after euthanasia. Serum was collected and measured for (**C**) HMGB1 and (**D**) IL-6. Extracellular-rich fluid from the injured or control paws was obtained using the EXPEL method, and levels of (**E**) HMGB1, (**F**) IL-6, and (**G**) CGRP were measured. *N* = 10 mice per group. Data are presented as mean ± SEM. Statistical significance was determined by one-way ANOVA followed by Tukey’s multiple comparisons test..n.s.: not significant, *: *P* < 0.05, **: *P* < 0.01, ***: *P* < 0.001, ****: *P* < 0.0001

**Fig. 2:**
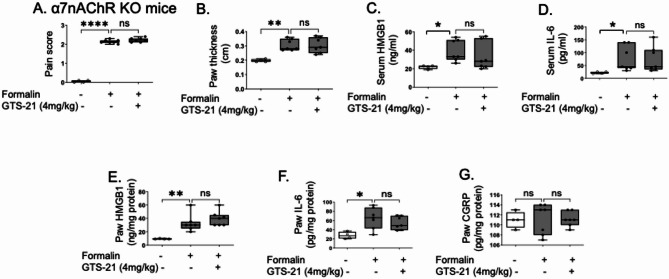
GTS-21 did not affect formalin-induced pain-like behavior in α7nAChR knockout mice. α7nAChR KO mice were administered GTS-21 (4 mg/kg) or vehicle (PBS) intraperitoneally 30 min before formalin injection (5%, 10 µl/paw) into the right hind paw. **A** Pain-like behavior was assessed 5 to 45 min post-injection. **B** Paw thickness was measured after euthanasia. Serum was collected and measured (**C**) HMGB1 and (**D**) IL-6. Extracellular-rich fluid from the injured or control paws was obtained using the EXPEL method, and levels of (**E**) HMGB1, (**F**) IL-6, and (**G**) CGRP were measured. *N* = 4–7 mice per group. Data are presented as mean ± SEM. Statistical significance was determined by one-way ANOVA followed by Tukey’s multiple comparisons test (A,G). Kruskal–Wallis test was used followed by Dunn’s post-hoc test (B-E). n.s.: not significant, *: *P* < 0.05, **: *P* < 0.01, ****: *P* < 0.0001

As an initial step to investigate the role of cholinergic agonists in HMGB1-mediated neuronal inflammation, we used a formalin-induced allodynia mouse model. Wild type (C57BL/6) mice pre-treated with the α7nAChR selective agonist GTS-21 (4 mg/kg) showed 40% reduction in formalin-induced pain scores (Fig. 1A) and swelling (Fig. 1B) compared to controls. Although no difference was detected in serum levels of HMGB1 and IL-6 (Fig. 1C-D), GTS-21 significantly reduced local HMGB1 levels in inflamed paws (Fig. 1E-F). In contrast calcitonin gene-related peptide (CGRP) levels in the paw tissue were not altered by the treatment (Fig. 1G). In α7nAChR KO mice, administration of formalin to the paw resulted in a pain-like behavior response and swelling like those observed in wild type mice (Fig. 2A-B). Although no significant elevation in serum HMGB1 and IL-6 levels following formalin paw injection (Fig. 2C-D). HMGB1 and IL-6 levels were elevated in the formalin-induced inflamed paws of both α7nAChR KO and wild type mice (Fig. 2E-F). In contrast, GTS-21 failed to reduce pain-like behavior, swelling, HMGB1 and IL-6 levels in the formalin model in α7nAChR KO mice (Fig. 2A-F), demonstrating that α7nAChR is essential for the anti-inflammatory and analgesic effects of GTS-21 in this model. As with wild type mice, GTS-21 treatment did not alter CGRP levels in the paw tissue in α7nAChR KO mice (Fig. 2G).

**Fig. 3:**
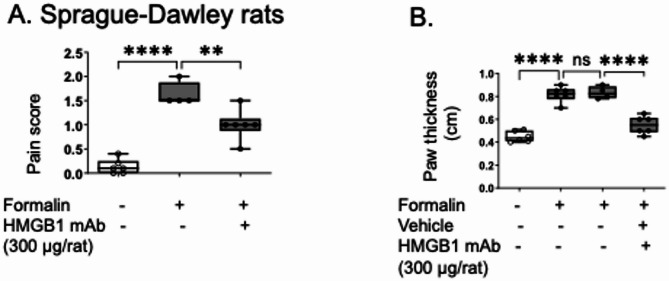
HMGB1 mAb 2g7 ameliorated formalin-induced pain-like behavior in rats. Sprague–Dawley rats received HMGB1 mAb 2g7 (300 µg/rat) or vehicle (PBS) intraperitoneally 30 min before formalin injection (5%, 50 µl/paw) into the right hind paw. **A** Pain-like behavior was assessed 5–45 min after formalin injection and (**B**) Paw thickness was measured following euthanasia. *N* = 4–6 rats per group. Data are represented as mean ± SEM. Statistical significance was determined by one-way ANOVA followed by Tukey’s multiple comparisons test. n.s.: not significant. **: *P* < 0.01, ****: *P* < 0.0001

To verify the findings observed using mice, next we used Sprague–Dawley rats. They were treated with the neutralizing anti-HMGB1 monoclonal antibody (2g7 intraperitoneal injection) prior to formalin injection into the hind paw and exhibited reduced pain scores and paw swelling (Fig. 3A-B), further confirming the role of HMGB1 in formalin-induced pain-like behavior.

### GTS-21 ameliorated optogenetically-induced mechanical allodynia in Vglut2-Cre/ChR2-eYFP mice

**Fig. 4:**
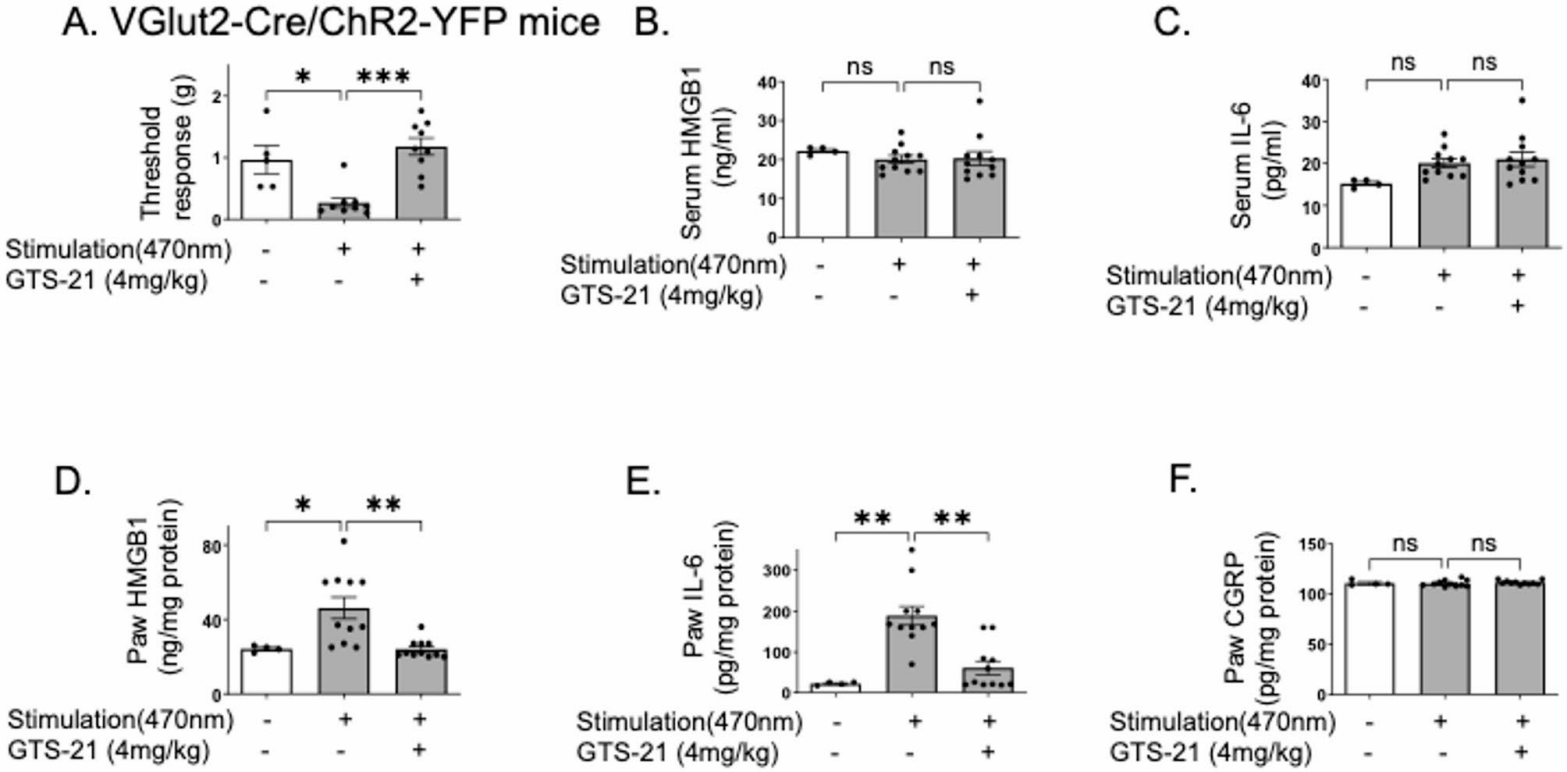
GTS-21 ameliorated optogenetically-induced nociceptive behavior in Vglut2-ChR2-YFP mice. VGlut2-ChR2-YFP mice were anesthetized with isoflurane (1.5–2%) and subjected to optogenetic stimulation (470 nm, 4.7 mW, 3 Hz, 20% duty cycle) on the right hind paw for 15 min, followed by intraperitoneal injection GTS-21 (4 mg/kg, in 100 μl volume) or vehicle (PBS). **A** Mechanical allodynia was assessed with von Frey filaments and analyzed using Dixon’s up down method to determine the threshold response. Serum and paw tissues were collected 5 h post-stimulation, and levels of (**B**) serum HMGB1 (**C**) serum IL-6, (**D**) paw HMGB1, (**E**) paw IL-6, and (**F**) paw CGRP were measured. *N* = 4–9 mice per group. Data are presented as mean ± SEM. Statistical significance was determined by one-way ANOVA followed by Tukey’s multiple comparisons test (B,C,D,F). Kruskal–Wallis test was used followed by Dunn’s post-hoc test (A,E). n.s.: not significant, *: *P* < 0.05, **: *P* < 0.01, ***: *P* < 0.001

These findings were replicated in an acute optogenetic activation of nociceptors model using transgenic Vglut2-Cre/ChR2-eYFP mice, in which neurons express channelrhodopsin-2 (ChR2) which can be activated by exposure to 470 nm light. We performed immunohistochemistry experiments to confirm the ChR2-eYFP expression on nociceptors (supplement Fig. 1A-D). In cultured DRGs isolated from Vglut2-Cre/ChR2-eYFP mice, strong eYFP labeling was observed (supplement Fig. 1B) and greatly overlapped with eYFP neurons stained with NeuN (supplement Fig. 1B-D). As shown in Fig. 4, optogenetic stimulation of the hind paw induced mechanical allodynia, which was significantly reduced by GTS-21 treatment in Vglut2-Cre/ChR2-eYFP mice (Fig. 4A), without altering serum HMGB1 and IL-6 levels (Fig. 4B-C). As expected, administration of GTS-21 significantly attenuated optogenetically-induced HMGB1 and IL-6 release locally in the inflamed paw (Fig. 4D-E). Despite the established role of CGRP in neuroinflammatory pain in some models, we did not observe significant changes in tissue levels of CGRP after optogenetic activation or GTS-21 treatment (Fig. 4F). Together, these observations indicate that the cholinergic agonist GTS-21 modulated the release of HMGB1, but not CGRP, from nociceptors.

### Cholinergic agonists significantly reduced capsaicin-induced release of HMGB1, but not CGRP and substance P, in cultured DRGs from wild type mice

**Fig. 5:**
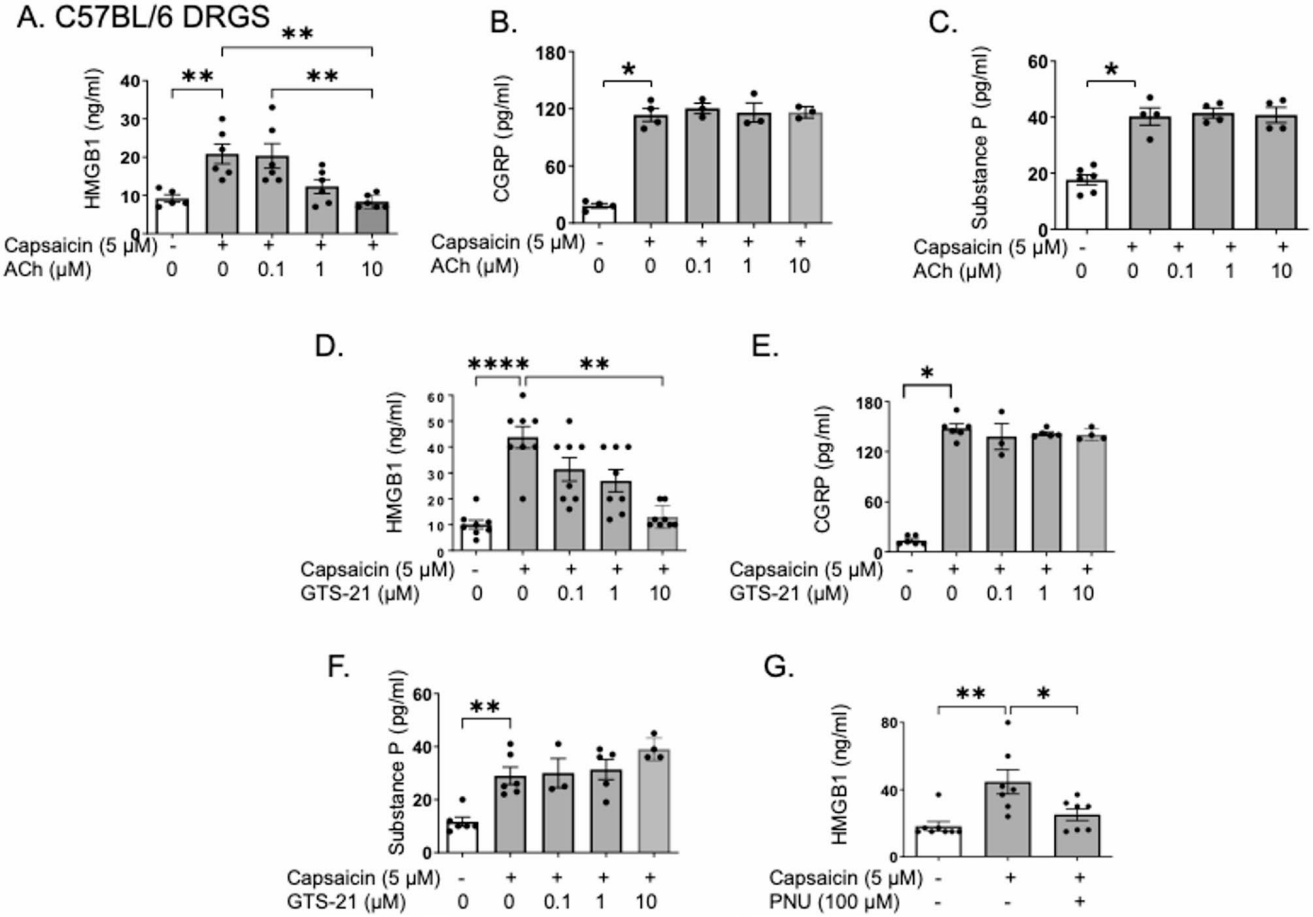
Cholinergic agonists reduced capsaicin-induced HMGB1, but not CGRP and substance P release, in cultured DRGs from wild type mice. DRGs isolated from C57BL/6 mice in culture dishes were pre-incubated with acetylcholine (0.1–10 µM) plus pyridostigmine bromide (1 µM, to inhibit acetylcholine degradation) for 1 h, stimulated with capsaicin (5 μM) or vehicle, and supernatant was collected after 2 h for measurement of (**A**) HMGB1, (**B**) CGRP and (**C**) substance P. In separate experiments, DRGs from C57BL/6 mice in culture dishes were pre-incubated with GTS-21 (0.1–10 µM) for 1 h, followed by stimulation with capsaicin (5 μM). Cell supernatants were collected 2 h post stimulation and used for (**D**) HMGB1, (**E**) CGRP and (**F**) substance P measurement. In separate experiments, DRGs isolated from C57BL/6 mice in culture dishes were pre-incubated with 100 μM PNU-282987** (**or vehicle PBS) for 1 h, stimulated with capsaicin (5 μM) and supernatants were collected after 2 h for measurements of (**G**) HMGB1 measurement. *N* = 3–8 per group. Data are presented as mean ± SEM. Statistical significance was determined by one-way ANOVA followed by Tukey’s multiple comparisons test. *: *P* < 0.05, **: *P* < 0.01, ****: *P* < 0.0001

After demonstrating that cholinergic agonists reduced HMGB1 levels in inflammatory pain models, we further investigated the role of α7nAChR in cholinergic agonist-mediated inhibition of HMGB1 release. We thus performed in vitro assays using DRG sensory neurons isolated from wildtype or α7nAChR KO mice. DRGs were pre-incubated with acetylcholine, followed by stimulation with capsaicin, a TRPV1 agonist that activates nociceptors. Capsaicin-induced HMGB1 release was dose-dependently inhibited by the addition of acetylcholine (Fig. 5A). In addition to HMGB1, we examined whether cholinergic agonists affected the release of other neuron-derived mediators, such as CGRP and substance P. Notably, cholinergic agonists did not affect the release of either CGRP or substance P (Fig. 5B-C). Importantly, cell viability assessment revealed that reduced HMGB1 release was not caused by cell death, as indicated by uninfluenced LDH release, a marker for cell membrane disruption (Kumar 2018) (Supplement Fig.2A). The results for HMGB1, CGRP, and substance P release after acetylcholine treatment were recapitulated using GTS-21. Exposure to GTS-21 also significantly and dose-dependently reduced capsaicin-induced HMGB1 release from cultured DRG nociceptors of wild type mice, without affecting the release of CGRP or substance P (Fig. 5D-F), and without altering LDH release (Supplement Fig. 2B). Furthermore, co-cultures with the highly specific α7nAChR agonist PNU-282987 generated similar results (Fig. 5G). Since HMGB1 lacks a secretory signal sequence and does not follow the classical vesicular release pathway, these observations are in line with reports showing that HMGB1 release occurs via a lysosome/endosome/autophagosome-dependent mechanism (Blakely and Edwards 2012; Kaya et al. 2023; Mao et al. 2022; Yang 2021).

### Cholinergic agonists did not reduce capsaicin-induced HMGB1, CGRP and substance P release in cultured DRGs from α7nAChR KO mice

**Fig. 6:**
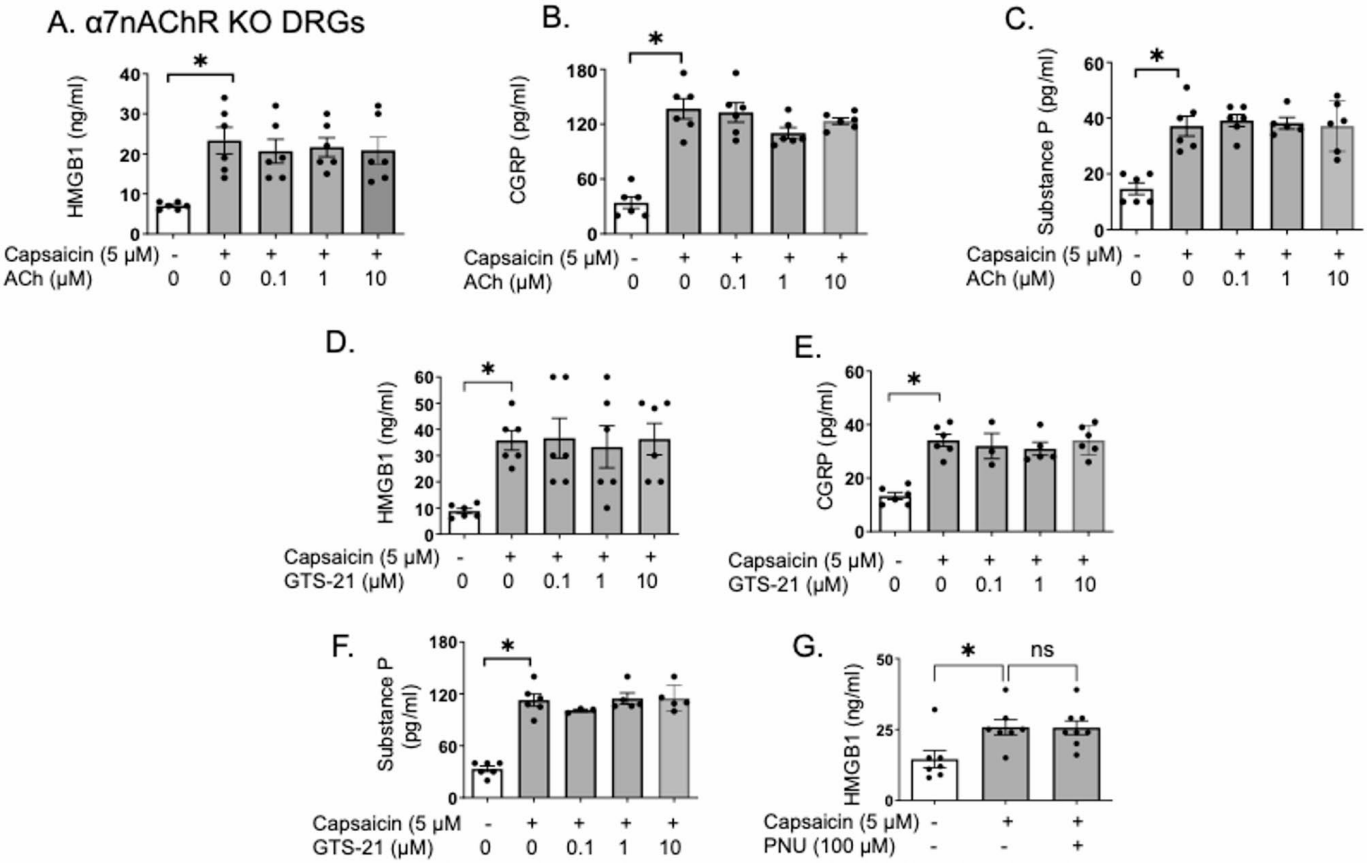
Cholinergic agonists did not reduce capsaicin-induced HMGB1, CGRP and substance P release in cultured DRGs from α7nAChR KO mice. DRGs from α7nAChR KO mice were cultured in dishes and pre-incubated with acetylcholine (0.1–10 µM) plus pyridostigmine bromide (1 µM) for 1 h, followed by stimulation with capsaicin (5 μM) or vehicle, and supernatants were collected after 2 h for measurement of (**A**) HMGB1, (**B**) CGRP, and (**C**) substance P. In separate experiments, DRGs in culture dishes were pre-incubated with GTS-21 (0.1–10 µM) or PNU-282987 (100 µM) for 1 h, stimulated with capsaicin (5 μM). Supernatants were collected 2 h post-stimulation for measurements of (**D**, **G**) HMGB1, (E) CGRP and (**F**) substance P release. *N* = 5–8 per group. Data are presented as mean ± SEM. Statistical significance was determined by one-way ANOVA followed by Tukey’s multiple comparisons test. n.s.: not significant. *: *P* < 0.05

To explore the role of neuronal α7nAChR in acetylcholine-induced inhibition of HMGB1 release in capsaicin-activated nociceptors, we next used DRGs obtained from α7nAChR KO mice. Consistent with our in vivo findings (Fig. 2), capsaicin stimulation of DRGs from α7nAChR KO mice led to an increase in HMGB1 release, which was not significantly altered by the addition of acetylcholine (Fig. 6A). Like wild type DRGs (Fig. 5), capsaicin-induced CGRP and substance P release was not significantly affected in α7nAChR-deficient DRGs by acetylcholine exposure (Fig. 6B-C); GTS-21 as well as PNU-282987 failed to inhibit capsaicin-induced HMGB1, CGRP and substance P release in DRGs of α7nAChR KO mice (Fig. 6D-G), further highlighting the necessity of α7nAChR in this process.

### Cholinergic agonists blocked optogenetically-induced release of HMGB1 but not CGRP or substance P in cultured Vglut2-Cre/ChR2-eYFP DRGs

**Fig. 7:**
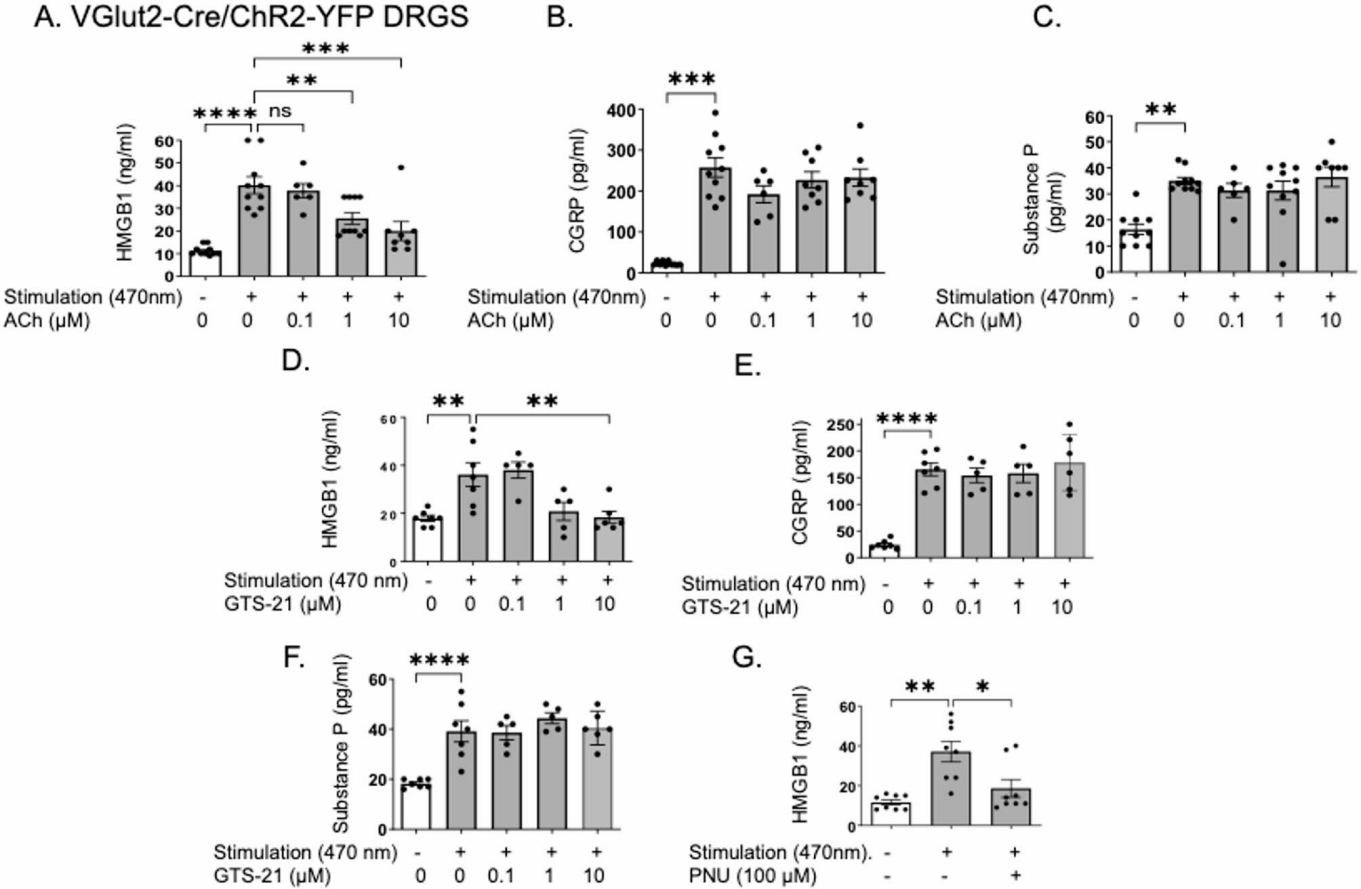
Cholinergic agonists reduced optogenetically-induced HMGB1 release, but did not affect CGRP or substance P release, in cultured DRGs from Vglut2-ChR2-YFP mice. DRGs from Vglut2-ChR2-YFP mice were pre-incubated with acetylcholine (0.1–10 µM) plus pyridostigmine bromide (1 µM) for 1 h, followed by light stimulation (wave length 470 nm) for 15 min. Cell supernatants were collected 2 h post-stimulation for measurements of (**A**) HMGB1, (**B**) CGRP and (**C**) substance P released. In separate experiments, DRGs were pre-incubated with GTS-21 (0.1–10 µM) or PNU-282987 (100 µM) for 1 h, followed by light stimulation (wave length 470 nm) for 15 min. Cell supernatants were collected 2 h later for measurements of (**D**, **G**) HMGB1, (**E**) CGRP, and (**F**) substance P. *N* = 5–10 per group. Data are presented as mean ± SEM. Statistical significance was determined by one-way ANOVA followed by Tukey’s multiple comparisons test. n.s.: not significant. *: *P* < 0.05, **: *P* < 0.01, ***: *P* < 0.001, ****: *P* < 0.0001

To further investigate the effects of cholinergic agonists, we developed an in vitro assay using DRG sensory neurons expressing channelrhodopsin-2 (ChR2) enabling specific activation using light stimulation (Yang 2021). Our findings revealed that similar to the capsaicin model in wild type DRGs (Fig.5), acetylcholine significantly and dose-dependently reduced optogenetically evoked HMGB1 release in DRG nociceptors obtained from Vglut2-Cre/ChR2-eYFP mice, without affecting CGRP or substance P release (Fig. 7A-C). LDH levels remained unchanged across treatment, confirming that neither optogenetic stimulation nor exposure to the cholinergic agonists induced cell damage (Supplement Fig. 2C).

To corroborate the findings obtained with acetylcholine (Fig. 7A-C), we used GTS-21 or PNU-282987 to selectively activate α7nAChR and found that they also specifically inhibited optogenetically-elicited HMGB1 release (Fig. 7D,G), without affecting CGRP or substance P release (Fig. 7E-F).

### GTS-21 inhibited capsaicin-induced nuclear-to-cytoplasm translocation of HMGB1 in wildtype DRGs

**Fig. 8:**
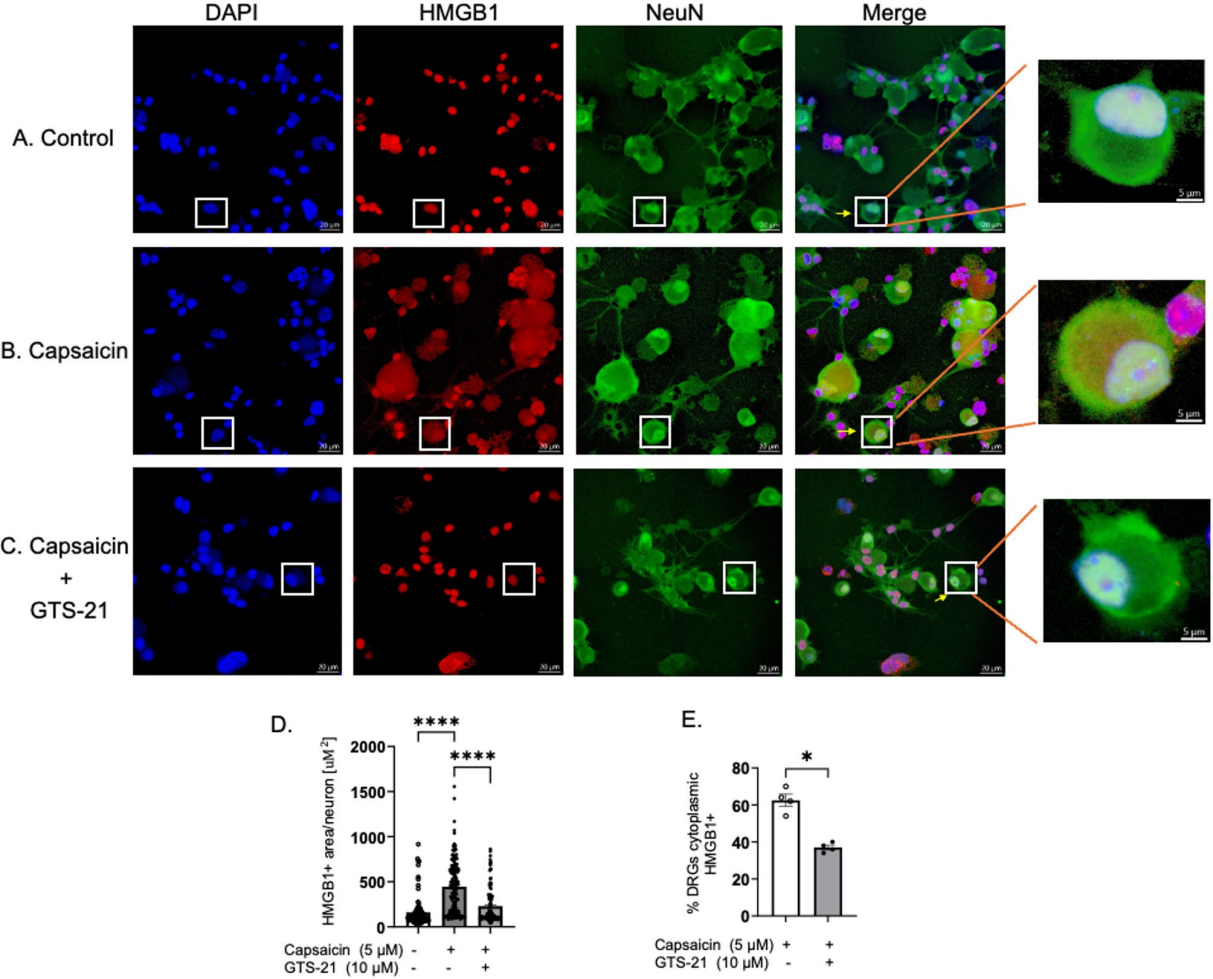
GTS-21 blunted capsaicin-induced nuclear-to-cytoplasm translocation of HMGB1 in wild type DRGs. DRGs were harvested from C57BL/6 mice and cultured for 48 h in plates with poly-L-lysine and laminin coated coverslips before treatment with vehicle, capsaicin (5 µM), or capsaicin plus GTS-21 (10 µM). After treatment, DRGs were fixed and stained with DAPI (blue) for nuclei, NeuN (green) for neurons, and HMGB1 (red) to observe HMGB1 translocation. Representative images for HMGB1, DAPI, and NeuN, along with their overlays. Control (**A**) capsaicin-treated (**B**), and capsaicin + GTS-21 (**C**) illustrate regions where HMGB1 translocation was quantified, with corresponding close-up images. Scale bars = 20 µm. For quantification, 150 DRGs per group were randomly selected from three representative tile scans (50 DRGs per scan). The area of HMGB1 + staining was traced using the spline tool in Zen Blue software (Zeiss Inc., White Plains, NY). (**D**) The total area of HMGB1 + staining and (**E**) the percentage of DRGs exhibiting cytoplasmic HMGB1 localization were quantified. Data are presented as mean ± SEM. Statistical significance was determined by one-way ANOVA followed by Tukey’s multiple comparisons test (**D**). Unpaired Student t test was used (**E**). *: *P* < 0.05, ****: *P* < 0.0001

Having demonstrated that cholinergic agonists reduced HMGB1 release from sensory neurons, we next investigated the mechanism underlying this inhibited release. HMGB1 is a nuclear protein, and its extracellular release depends upon its translocation from the nucleus to the cytoplasm as a key initial step, which has been demonstrated in immune and smooth muscle cells (Bonaldi et al. 2003; Kaya et al. 2023; Lu et al. 2012). Our results revealed that GTS-21 inhibited the nuclear HMGB1 export also in neurons (Fig.8). Quiescent sensory neurons predominantly retained HMGB1 in the nucleus (Fig. 8A). Following capsaicin exposure, significant nuclear HMGB1 translocation to the cytoplasm was observed in sensory neurons, indicating an active mobilization of the nuclear HMGB1 prior to its extracellular release (Fig. 8B). In contrast, pretreatment with GTS-21 significantly inhibited capsaicin-induced nuclear-to-cytoplasmic translocation of HMGB1 in DRG sensory neurons. Most HMGB1 was retained in the nucleus, resulting in reduced extracellular release (Fig. 8C). Quantitative analyses confirmed a highly significant reduction in nuclear-to-cytoplasm HMGB1 translocation following GTS-21 treatment (Fig. 8D-E).

## Discussion

Our study demonstrates that cholinergic agonists effectively reduced pain-like behavior and inflammation in models of allodynia. These effects were attained via α7nAChR-mediated signaling, which inhibited the release of HMGB1 from sensory nociceptors. Specifically, the α7nAChR-selective agonist GTS-21 prevented the nuclear-to-cytoplasmic translocation of HMGB1, thereby blocking its extracellular release. This mechanism highlights the targeted action of cholinergic agonists on upstream pathways that drive HMGB1-dependent inflammation and nociceptive behavior. Recent studies revealing the structure of human α7nAChRs have confirmed their link to reduced nociceptive behavior, providing a strong starting point for developing targeted treatments (Tao et al. 2025).

HMGB1 and acetylcholine are ancient molecules predating the evolution of the nervous system by over 400 million years, signifying their fundamental roles in physiological processes (Changeux 2018; Sharman et al. 1997). These molecules function in a yin-yang manner to maintain inflammatory balance: HMGB1 promotes inflammation, while acetylcholine, predominantly via α7nAChR-mediated pathways, counters HMGB1-driven inflammatory responses. This balance is crucial for maintaining homeostasis during inflammatory responses, with acetylcholine serving as a counter-regulatory mechanism to prevent excessive inflammation (Kalashnyk et al. 2020; Keever et al. 2023; Xu et al. 2021). The dual ancient roles of HMGB1 and acetylcholine highlight their evolutionary importance in maintaining inflammatory balance, with the α7nAChR-dependent cholinergic pathway representing a key mediator of anti-inflammatory control.

The mechanism underlying active HMGB1 release in non-neuronal cells (macrophage, endothelial, and smooth muscle) have been partially identified; however, that governing HMGB1 release from nociceptors remain unknown. Notably, the kinetics of HMGB1 release from activated nociceptors are strikingly different from those observed in all other studied cell types (Liu et al. 2018; Lu et al. 2014c; Wei et al. 2022; Zhou et al. 2018). While most cells require several hours to release HMGB1 extracellularly following stimulation, nociceptors are remarkably rapid in initiating active HMGB1 release in less than one hour as we previously reported (Yang 2021). This discovery is both novel and significant, particularly in light of our finding that α7nAChR signaling can inhibit this rapid HMGB1 release from nociceptors.

One potentially clinically important aspect of this rapid release is its relevance in the context of severe trauma. In a study by Ottestad et al., two distinct HMGB1 release phases were identified following trauma (Ottestad et al. 2019). The first, an initial exponential decay phase with a half-life of 26 min, was not correlated with clinical outcomes. In contrast, a second wave peaking between 3–6 h post-trauma was strongly predictive of poor clinical outcomes in severely injured and physiologically compromised patients. The initial HMGB1 peak is likely due to passive release from necrotic tissue and therefore cannot be a therapeutic target. Although the cellular source of the critical second HMGB1 peak remains unidentified, based on current knowledge, nociceptors are the most plausible contributors; being the only known cell type capable of actively releasing HMGB1 within a time frame consistent with the 3–6 h post-trauma peak. By comparison, HMGB1 release from macrophages following LPS stimulation occurs over 8–16 h (Wang et al. 1999) Gazzar et al.^2007^.

Therefore, our finding that α7nAChR signaling can suppress HMGB1 release from nociceptors may have important implications for future therapeutic strategies in trauma care.

Although the precise mechanisms by which HMGB1 is released from activated nociceptors, and how α7nAChR inhibits this process remain to be elucidated, we have demonstrated that one critical step which can be inhibited by α7nAChR activation prevents the translocation of nuclear HMGB1 into the cytosol.

The release mechanisms of the neuropeptide substance P and CGRP appear to differ from that of HMGB1. Our observations indicate that cholinergic agonists did not significantly affect the release of CGRP or substance P, in contrast to their clear influence on HMGB1 release (Figs. 5B,C,E,F). Neuropeptide release typically follows the classical secretory granule pathway, which involves vesicular exocytosis from neurons (Blakely and Edwards 2012). In contrast, the mechanisms underlying neuronal release of HMGB1 are not yet fully elucidated. In macrophages, HMGB1 secretion has been shown to involve autophagosome/endosome-based pathways and is mediated via intracellular vesicles such as secretory lysosomes (Gardella et al. 2002), microvesicles, and multivesicular bodies (Kim et al. 2021; New and Thomas 2019).

Additionally, Bonaldi et al. demonstrated that nuclear HMGB1 must undergo acetylation to exit the nucleus (Bonaldi et al. 2003). Several studies have shown that histone deacetylases (HDACs), including SIRT1, counteract HMGB1 release by removing acetyl groups from nuclear HMGB1 (Contis-Montes 2019; Evankovich et al. 2010; He et al. 2013; Shen et al. 2025; Sixto-Lopez 2020; Walko et al. 2015; Zou and Crews 2014). The deacetylase-mediated inhibition of HMGB1 translocation and release has been documented in different cell types including neurons (Zou and Crews 2014).

In line with our findings, numerous reports have confirmed that α7nAChR stimulation inhibits HMGB1 extracellular release in various cell types (Mei et al. 2018; Sitapara et al. 2020b; Wang et al. 2004, 2012; Wazea et al. 2018). The fact that both α7nAChR signaling and HDAC activation led to nuclear retention of HMGB1 raises the question of a potential functional link between these mechanisms. Two independent studies support such a connection, demonstrating that α7nAChR activation enhances deacetylase activity via SIRT1 (Li et al. 2016)and SIRT3 (Li et al. 2019). Based on this, we speculate that α7nAChR-mediated signaling may increase intranuclear deacetylase activity in nociceptors, thereby promoting HMGB1 retention in the nucleus and accounting for our observed results. Notably, SIRT1 is known for its anti-inflammatory properties and plays a key role in regulating inflammation. Its expression declines with age, contributing to age-associated diseases such as Alzheimer's, Parkinson's, and Huntington's disease (Wang et al. 2014).

While DRGs are not directly innervated by the vagus nerve, we show that acetylcholine can significantly influence DRG function. However, the source of acetylcholine for DRG modulation remains unknown. Mobile non-neuronal cells, such as choline acetyltransferase-expressing (ChAT positive) T lymphocytes, can serve as a source of endogenous acetylcholine signaling (Olofsson et al. 2016; Rosas-Ballina et al. 2011). Studies have demonstrated that ChAT-expressing T cells deliver acetylcholine to distant sites influencing several distinct pathways including the cholinergic anti-inflammatory mechanism (Cox et al. 2019; Ramirez 2019; Rosas-Ballina et al. 2011; Tarnawski et al. 2023; Willemze et al. 2019) Shavva et al.^2024^. Additionally, recent findings by Gabalski et al. (2024) indicate that circulating ChAT molecules, presumably available to all tissues, act as anti-inflammatory agents (Gabalski et al. 2024). Moreover, Corsetti et al. showed that DRGs can synthesize and store acetylcholine in vesicles by demonstrating that rat DRG neurons express key cholinergic markers, including mRNA transcripts for choline acetyltransferase (ChAT) and vesicular acetylcholine transporter (VAChT) (Corsetti 2021). Together, these findings suggest that acetylcholine can potentially be supplied via multiple routes to DRGs to attenuate inflammatory signaling. Further research must be done to elucidate which sources of cholinergic signaling to DRGs are significant and how these signals reach the location of DRGs.

In summary, our findings indicate that α7nAChR selective agonists such as GTS-21 and PNU-282987 inhibit the translocation of HMGB1 from nucleus to cytoplasm in neurons and therefore its release. Moreover, we show that these cholinergic agonists prevent neuronal extracellular HMGB1 release and subsequent inflammation, mirroring our prior observations obtained from neuronal HMGB1 KO mice (Yang 2021). The cholinergic anti-inflammatory pathway, primarily regulated by the vagus nerve, involves acetylcholine signaling via α7nAChR on immune cells, which blunts pro-inflammatory cytokine release and inflammation. Extending the role of cholinergic signaling to the regulation of HMGB1 release in neurons elucidates a new form of communication between the nervous and immune systems, paving the way for novel therapeutic strategies. Approaches directly targeting HMGB1 with antibodies have faced challenges due to HMGB1’s tendency to bind to diverse molecules, making it rarely found extracellularly in isolation and with reasonable blocking attachment sites. Modulation of HMGB1 release via α7nAChR offers an alternative approach to achieve this therapeutic goal, circumventing its adhesive properties (Koopman et al. 2016; Mao et al. 2022; Yang et al. 2019). Finally, it is interesting that in vivo α7nAChR agonists do not inhibit the release of CGRP and substance P, yet block pain-like behavior. In light of recent findings showing that dual knockout of CGRP and substance P does not affect the development of nociceptor behavior in number of preclinical models (MacDonald 2025), our findings suggest HMGB1 is a key player in pain pathways and that inhibiting its release using α7nAChR agonists may be a promising analgesic approach.

## Supplementary Information

Supplementary Material 1: Supplement figure 1: DRGs isolated from Vglut2-ChR2-YFPmice were cultured in 24 well plates with poly-L-lysine and laminin coated coverslips for 48 hours. DRGs were fixed and stained with DAPIfor nuclei, greenand NeuNfor neurons and the overlays. Scale bar=100 um. Data are representative of 5 images from N=4 mice, 1-2 sections per mouse.

Supplementary Material 2: Supplement figure 2: Cholinergic agonists did not significantly alter LDH release in activated DRGs from wild type mice. DRGs isolated from C57BL/6 mice in culture dishes were pre-incubated with acetylcholineplus pyridostigmine bromidefor 1 hour, stimulated with capsaicin, and supernatant was collected after 2 hours for measurement of LDH. A cell lysate was included as a positive control for LDH content. In separate experiments, DRGs from C57BL/6 mice in culture dishes were pre-incubated with GTS-21for 1 hour, followed by stimulation with capsaicin. Cell supernatants were collected 2 hours post stimulation for LDH measurement. A cell lysate was included as a positive control for LDH content.DRGs from Vglut2-ChR2-YFPmice were pre-incubated with acetylcholineplus pyridostigmine bromidefor 1 hour, followed by light stimulationfor 15 minutes. Cell supernatants were collected 2 hours post-stimulation for measurements of LDH released. A cell lysate was included as a positive control for LDH content. N = 3-6 per group. Data are presented as mean ± SEM.

## Data Availability

The datasets analyzed during the current study are available from the corresponding author on reasonable request.

## Abbreviations

α7nAChR: α7 Nicotinic acetylcholine receptor
DRG: Dorsal root ganglia
HMGB1: High mobility group box 1
IP: Intraperitoneal
KO: Knockout
ELISA: Enzyme-linked immunosorbent assay
PBS: Phosphate buffered saline
TNF: Tumor necrosis factor
IL: Interleukin
ANOVA: Analysis of variance
DAPI: 4′,6-Diamidino-2-phenylindole
NeuN: Neuronal Nuclei
VGLUT2: Vesicular glutamate transporter 2
ChR2: Channelrhodopsin-2
CGRP: Calcitonin gene-related peptide
mAb: Monoclonal antibody
