# Supplementary figures and images for: Nociceptor α7nAChR activation blunts neuronal HMGB1 release and attenuates inflammation and nociceptive behavior
Source: Mol Med. 2025 Nov 3;31:324. doi: 10.1186/s10020-025-01387-z (PMC12581301; doi:10.1186/s10020-025-01387-z)

## Slide 1
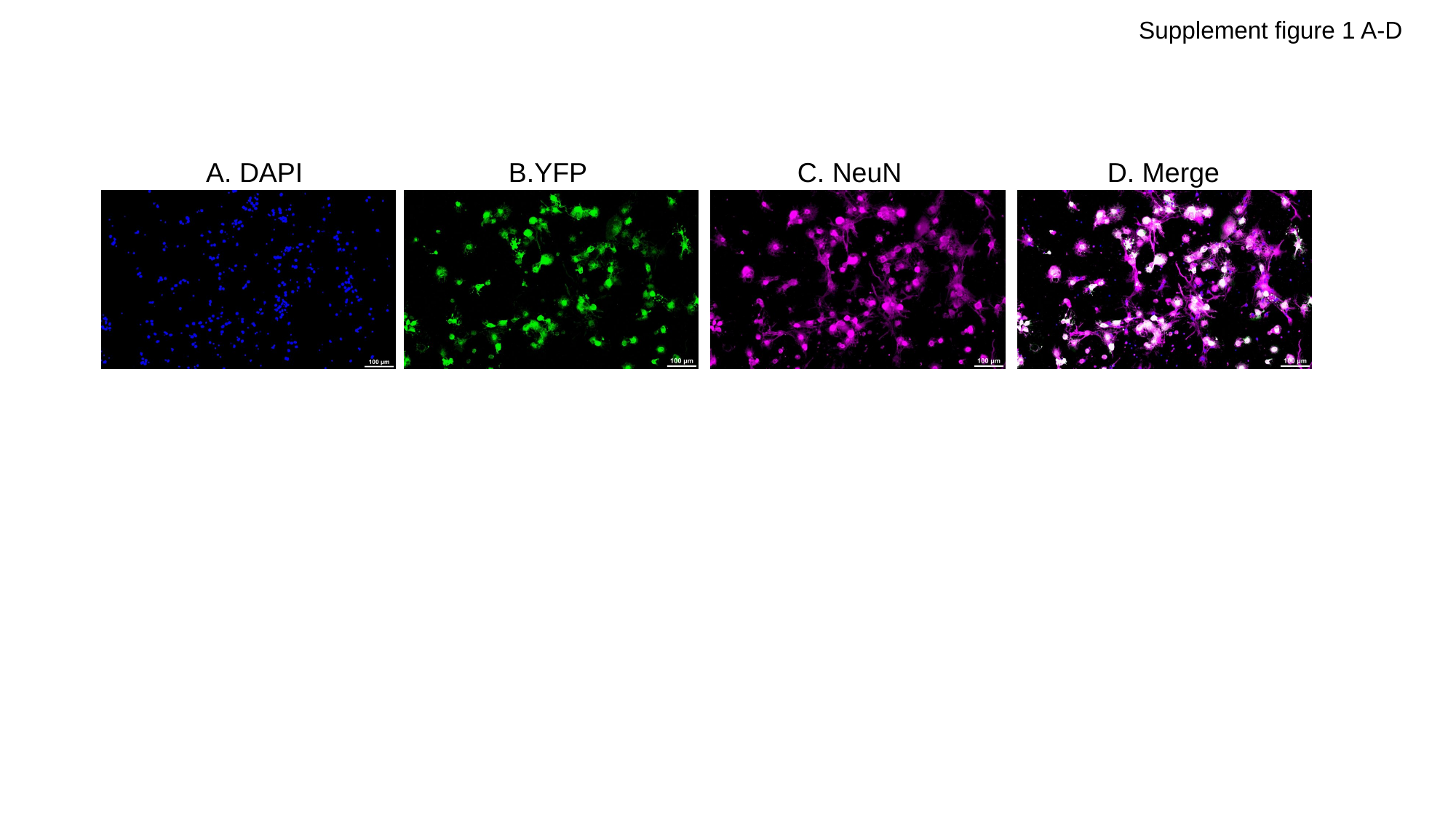

Supplement figure 1 A-D
A. DAPI B.YFP 	 C. NeuN D. Merge

Supplement: Supplementary file 1 — Supplementary Material 1: Supplement figure 1: DRGs isolated from Vglut2-ChR2-YFPmice were cultured in 24 well plates with poly-L-lysine and laminin coated coverslips for 48 hours. DRGs were fixed and stained with DAPIfor nuclei, greenand NeuNfor neurons and the overlays. Scale bar=100 um. Data are representative of 5 images from N=4 mice, 1-2 sections per mouse. [file 10020_2025_1387_MOESM1_ESM.pptx]
