# Supplementary material for: Nociceptor α7nAChR activation blunts neuronal HMGB1 release and attenuates inflammation and nociceptive behavior
Source: Mol Med. 2025 Nov 3;31:324. doi: 10.1186/s10020-025-01387-z (PMC12581301; doi:10.1186/s10020-025-01387-z)
Supplement: Supplementary file 2 — Supplementary Material 2: Supplement figure 2: Cholinergic agonists did not significantly alter LDH release in activated DRGs from wild type mice. DRGs isolated from C57BL/6 mice in culture dishes were pre-incubated with acetylcholineplus pyridostigmine bromidefor 1 hour, stimulated with capsaicin, and supernatant was collected after 2 hours for measurement of LDH. A cell lysate was included as a positive control for LDH content. In separate experiments, DRGs from C57BL/6 mice in culture dishes were pre-incubated with GTS-21for 1 hour, followed by stimulation with capsaicin. Cell supernatants were collected 2 hours post stimulation for LDH measurement. A cell lysate was included as a positive control for LDH content.DRGs from Vglut2-ChR2-YFPmice were pre-incubated with acetylcholineplus pyridostigmine bromidefor 1 hour, followed by light stimulationfor 15 minutes. Cell supernatants were collected 2 hours post-stimulation for measurements of LDH released. A cell lysate was included as a positive control for LDH content. N = 3-6 per group. Data are presented as mean ± SEM. [file 10020_2025_1387_MOESM2_ESM.pptx]

## Slide 1
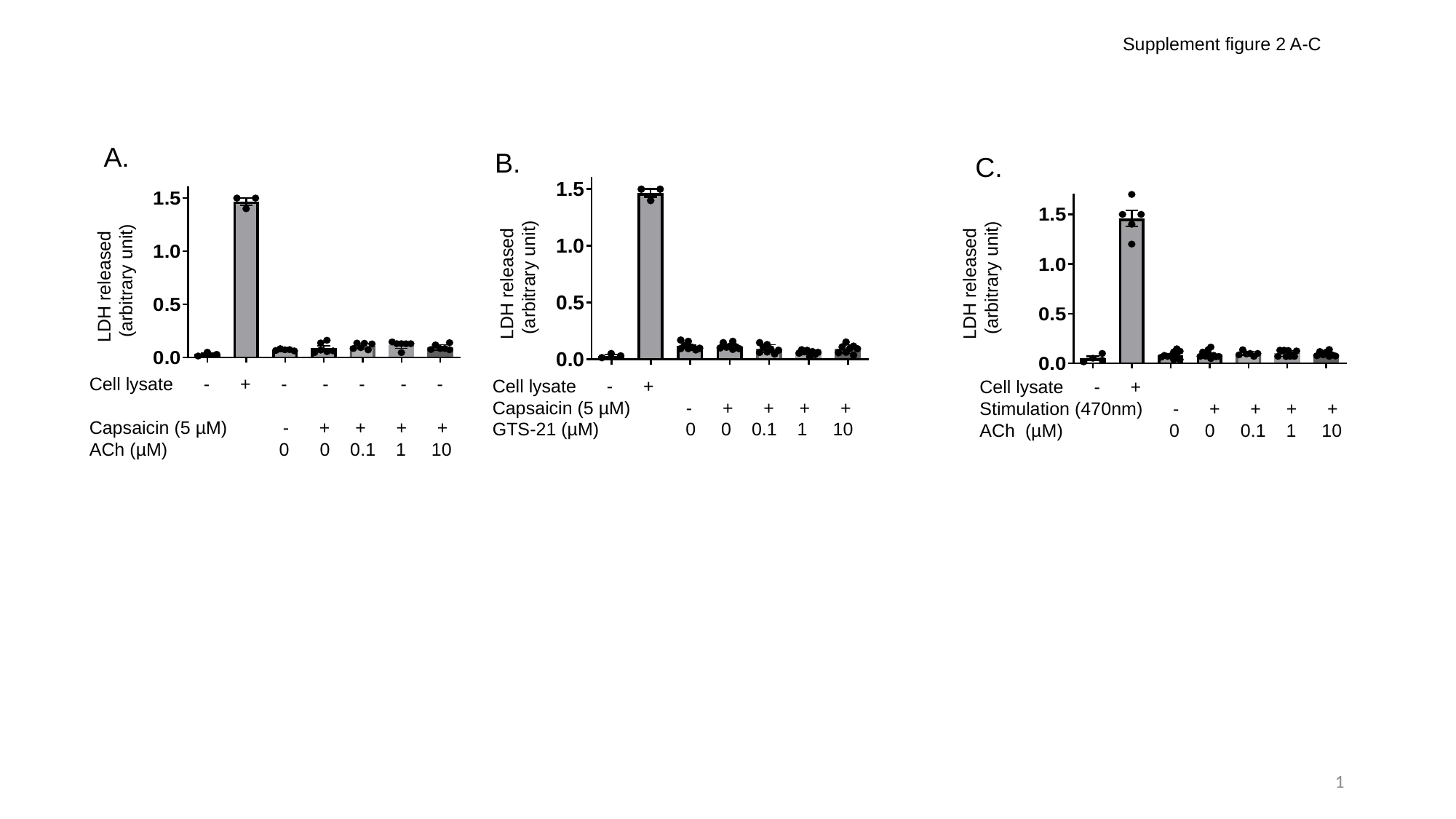

Supplement figure 2 A-C
A.
B.
C.
LDH released
 (arbitrary unit)
LDH released
 (arbitrary unit)
LDH released
 (arbitrary unit)
Cell lysate - + - - - - -
Capsaicin (5 µM) - + + + +
ACh (µM) 0 0 0.1 1 10
Cell lysate - +
Capsaicin (5 µM) - + + + +
GTS-21 (µM) 0 0 0.1 1 10
Cell lysate - +
Stimulation (470nm) - + + + +
ACh (µM) 0 0 0.1 1 10
1
